# Complete Genome Sequence and Pan-Genome Analysis of *Shewanella oncorhynchi* Z-P2, a Siderophore Putrebactin-Producing Bacterium

**DOI:** 10.3390/microorganisms11122961

**Published:** 2023-12-11

**Authors:** Ying Zhang, Mengjie Pan, Qiaoyun Wang, Lan Wang, Li Liao

**Affiliations:** 1 Key Laboratory of Cold Chain Logistics Technology for Agro-Product, Ministry of Agriculture and Rural Affairs/Institute of Agro-Product Processing and Nuclear Agricultural Technology, Hubei Academy of Agricultural Sciences, Wuhan 430064, China; zy920506@sina.com (Y.Z.); lilywang_2016@163.com (L.W.); 2 College of Food Science and Engineering, Wuhan Polytechnic University, Wuhan 430023, China; panmengjiefood@163.com (M.P.); wqy20210516@163.com (Q.W.)

**Keywords:** complete genome, pan-genome analysis, *Shewanella oncorhynchi*, siderophore, putrebactin

## Abstract

In this study, we reported the complete genome sequence of *Shewanella oncorhynchi* for the first time. *S. oncorhynchi* Z-P2 is a bacterium that produces the siderophore putrebactin. Its genome consists of a circular chromosome of 5,034,612 bp with a G + C content of 45.4%. A total of 4544 protein-coding genes, 109 tRNAs and 31 rRNAs were annotated by the RAST. Five non-ribosomal peptide synthetase (NRPS) and polyketide synthetase (PKS) gene clusters were identified by the antiSMASH analysis. The pan-genome analysis of Z-P2 and 10 *Shewanella putrefaciens* revealed 9228 pan-gene clusters and 2681 core gene clusters, with Z-P2 having 618 unique gene clusters. Additionally, the gene cluster involved in putrebactin biosynthesis in Z-P2 was annotated, and the mechanism of putrebactin biosynthesis was analyzed. The putrebactin produced by Z-P2 was detected using UPLC-MS analysis, with an [M + H]^+^ molecular ion at *m*/*z* 373.21. These findings provide valuable support for further research on the genetic engineering of putrebactin biosynthetic genes of Z-P2 and their potential applications.

## 1. Introduction

Iron (Fe) is an essential metal element for life on earth, playing a vital role in various biochemical reactions, such as energy production and biosynthesis [1,2]. However, iron is not bioavailable in aerobic environments because it exists in the form of insoluble ferric iron (Fe^3+^) [3]. In order to survive and compete, microorganisms and plants have developed siderophore-dependent iron uptake mechanisms to solubilize and capture iron [4]. Siderophores are low-molecular-weight organic chelators (500–1500 Da) that exhibit a strong affinity for insoluble Fe^3+^ and are biosynthesized under low-iron conditions by both prokaryotes and eukaryotes [5]. The biosynthesis of siderophores is generally catalyzed by two types of pathways—the non-ribosomal peptide synthetase (NRPS)-dependent pathway and NRPS-independent siderophore (NIS) pathway [6]. Siderophores are commonly classified into four types based on their chemical moieties, including hydroxamate, catecholate, carboxylate and mixed-type siderophores [1]. In addition, siderophores have great potential for application in various fields such as improving soil fertility and biocontrol in agriculture, decontaminating heavy metal-contaminated soils and water in the environment and treating some diseases in medicine [7].

*Shewanella* is the only genus in the family Shewanellaceae and consists of approximately 100 species of Gram-negative, facultative anaerobic and dissimilatory metal-reducing γ-proteobacteria [8,9]. These bacteria can be found in marine and freshwater environments. *Shewanella* has been extensively studied and utilized for the remediation of heavy metal-contaminated wastewaters and for electricity generation in microbial fuel cells [10]. However, *Shewanella* can also spoil aquatic products and act as pathogens for both fish and humans [8,11]. Moreover, numerous studies have indicated that the remarkable adaptive capabilities of *Shewanella* are attributed to its respiratory versatility, which requires a relatively high accumulation of iron for the synthesis of iron-containing proteins, iron–sulfur proteins and hemoproteins [12,13]. Therefore, it is important to investigate the iron uptake mechanisms and related genes in *Shewanella*.

In this study, we sequenced and analyzed the whole genome of the siderophore putrebactin-producing *Shewanella oncorhynchi* Z-P2. *S. oncorhynchi* is a novel species of the genus *Shewanella* and its complete genome sequence has never been reported [14]. Putrebactin is a cyclic hydroxamic acid-based 20-membered macrocycle siderophore produced by *Shewanella* [15]. *S. oncorhynchi* Z-P2 was isolated from the spoiled vacuum-packed aquatic products (crayfish) in Hubei, China, and has been deposited at the China General Microbiological Culture Collection Center (CGMCC) under accession number CGMCC No. 1.62135. The complete genome sequence of Z-P2 has been deposited at NCBI GenBank (CP132914). The pan-genome and core genome analysis of Z-P2 and *Shewanella putrefaciens*, which is the closely related specie of *S. oncorhynchi*, were performed at the whole-genome level for the first time. Additionally, the gene cluster involved in putrebactin biosynthesis in Z-P2 was annotated, and the mechanism of putrebactin biosynthesis was analyzed. This study has important implications for the understanding of the bacterial siderophore biosynthetic mechanism, particularly putrebactin produced by *Shewanella*. It also offers support for potential applications in drug production and further molecular biology research on related genes.

## 2. Materials and Methods

### 2.1. Genome Sequencing and Functional Annotation

Genomic DNA of *S. oncorhynchi* Z-P2 was extracted with the Bacterial DNA Kit (OMEGA BIO-TEK, Norcross, GA, USA). The genome was sequenced using the Nanopore PromethION platform and the Illumina NovaSeq PE150 at the Beijing Novogene Bioinformatics Technology Co., Ltd. (Beijing, China). Libraries for nanopore sequencing were constructed with an insert size of 10 kb and generated using NEBNext^®^ UltraTM DNA Library Prep Kit for Illumina (NEB, Houston, TN, USA). The resulting sequence was assembled with Unicycler [16]. Genome annotation was accomplished by rapid annotation using subsystem technology (RAST) [17]. Proteins of gene function annotation used the Clusters of Orthologous Groups (COG) database [18]. The metabolic pathways were annotated using the Kyoto Encyclopedia of Genes and Genomes (KEGG) pathway database (http://www.genome.jp/kegg, accessed on 18 August 2023). tRNA genes, rRNA genes and small nuclear RNAs (snRNA) were predicted by the tRNAscan-SE [19], rRNAmmer [20] and BLAST against the Rfam database [21], respectively. The Circos software [22] was used to display the circular genome. The phylogenetic tree of *S. oncorhynchi* Z-P2 based on the 16S rDNA gene sequences was constructed in MEGA 11 [23]. In addition, genomic islands were analyzed with IslandPath-DIOMB (Version 0.2) [24]. The virulence genes were analyzed by the Virulence Factors of Pathogenic Bacteria (VFDB) database [25]. CRISPRdigger (version 1.0) [26] was used for clustered, regularly interspaced, short palindromic repeat sequences (CRISPR) and CRISPR-associated (*cas*) gene prediction. Secondary metabolite biosynthesis gene clusters were conducted by the antiSMASH analysis [27].

### 2.2. Pan-Genome and Core Genome Analysis

The pan-genome and core genome of Z-P2 and 10 *S. putrefaciens* in NCBI (Table S1) were subjected to Integrated Prokaryotes Genome and Pan-genome Analysis (IPGA v1.09) [28] with the default settings. The genes of all filtered genomes were predicted using Prokka [29] for pan-genome analysis. All genes were annotated against the COG database and used to create pan-genome profiles using PanOCT [30]. The kSNP [31] was used for the whole-genome-based phylogenetic analysis, and genome-level syntenic analysis was performed using MUMmer [32]. The average nucleotide identity (ANI) values between each submitted genome pair were calculated.

### 2.3. Chromeazurol S (CAS) Assay Analysis of Siderophore

To assess siderophore production and secretion, Z-P2 was grown in M9 minimal salts medium (Zeye Biotechnology Co., Ltd., Shanghai, China) with 0.3% (*w*/*v*) casamino acids for 24 h at 35 °C, and cell-free culture supernatants were obtained by centrifugation (4000 rpm for 20 min at 4 °C). The CAS assay [33,34] was used for detection of siderophores in the supernatant. A total of 6 mL of 10 mM hexadecyltrimethyl ammonium bromide (HDTMA), 1.5 mL ferric iron solution (1 mM FeCl_3_·6H_2_O, 10 mM HCl), 7.5 mL of 2 mM aqueous CAS solution, 4.307 g anhydrous piperazine and 6.25 mL of 12 M hydrochloric acid were added to create 100 mL of CAS assay solution (filled with distilled water). A total of 3 mL of supernatant was mixed with 3 mL of the CAS assay solution and allowed to react at room temperature for 1 h. The absorbance of the mixture was measured at 630 nm (Spectrophotometer UV-5100, Shanghai Metash Instruments Co., Ltd., Shanghai, China). The measurement was repeated three times and the average value was taken. The siderophores relative content within the supernatant was expressed as siderophore production units (%), which were calculated according to the following formula: siderophore production units (%) = (Ar − As)/Ar × 100. Ar = absorbance of reference (CAS solution and uninoculated broth) and As = absorbance of mixture.

### 2.4. Mass Spectrometry Analysis of Siderophore

The obtained crude extract from the Z-P2 culture was separated by macroporous resin XAD-16 (AMBERLITE™, Supelco, Bellefonte, PA, USA) and collected fractions were analyzed by UPLC-MS. The Q Exactive-Mass spectrometer (Thermo Scientific, Sunnyvale, CA, USA) was operated in positive ion mode and connected to an Ultimate 3000 UHPLC (Dinonex, Thermo Scientific, Sunnyvale, CA, USA) equipped with an ACQUITY UPLC BEH C18 column (2.1 × 100 mm, 1.7 μm) (Waters, Milford, MA, USA). The mobile phases were A = water (0.1% formic acid) and B = acetonitrile (0.1% formic acid). The gradient elution program was as follows: T_0min_: B = 5%, T_1min_: B = 5%, T_10min_: B = 25%, T_15min_: B = 95%, T_18min_: B = 95% and T_20min_: B = 5%. The flow rate was 0.3 mL/min. The MS capillary temperature was maintained at 320 °C with an S-lens RF level at 50. The MS spectra collection was recorded at 7000 resolution with the scan range of 50 to 500 *m*/*z*.

## 3. Results and Discussion

### 3.1. Genome Features of S. oncorhynchi Z-P2

**Figure 1:**
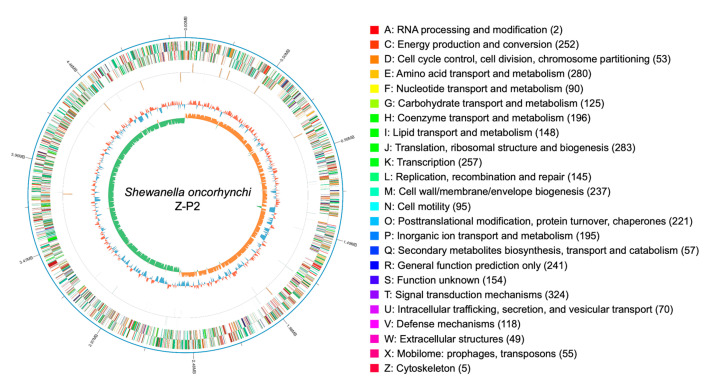
Circular genome map of *S. oncorhynchi* Z-P2. From outer to the inner: ring 1 represents genome size; ring 2 represents forward and reverse chains of coding DNA sequences (CDSs) colored according to COG categories; ring 3 represents forward and reverse chains of non-coding RNA (ncRNA) genes; ring 4 represents the G + C content (red, higher than the average; blue, lower than the average); ring 5 represents the GC skew (orange, positive skew; green, negative skew). Numbers in parentheses represent the count of genes with this functional type of COG.

**Figure 2:**
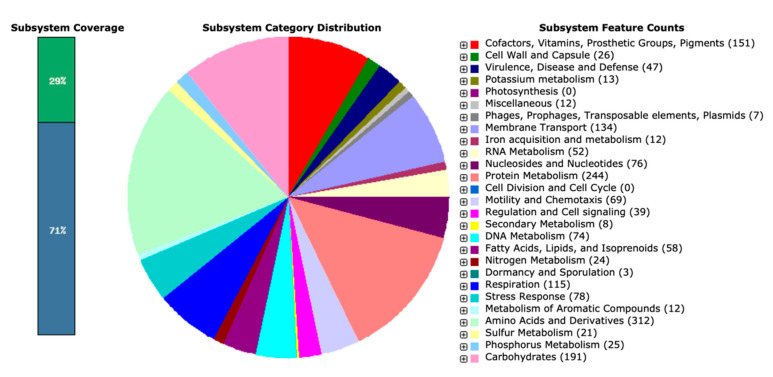
RAST subsystem distribution of *S. oncorhynchi* Z-P2.

**Figure 3:**
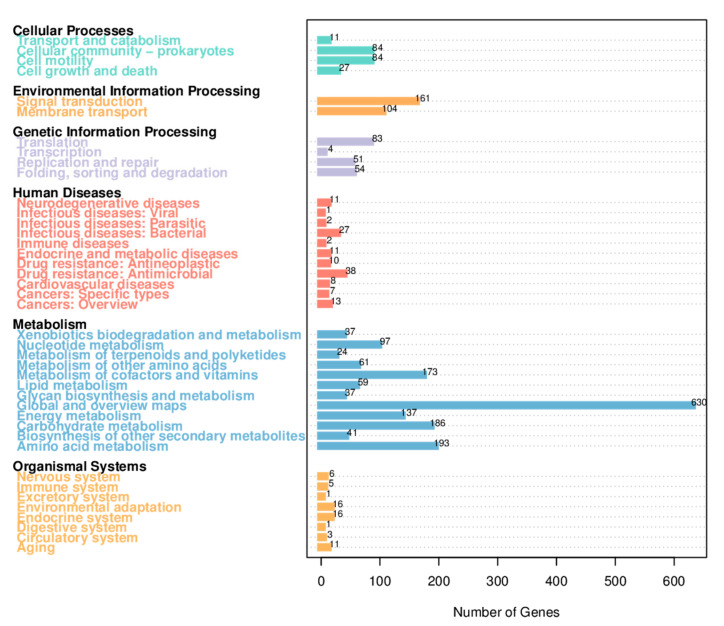
KEGG pathways annotation of *S. oncorhynchi* Z-P2. The level 1 pathways (in black) and level 2 pathways (in different color according to level 1 categories) were described on the left.

**Figure 4:**
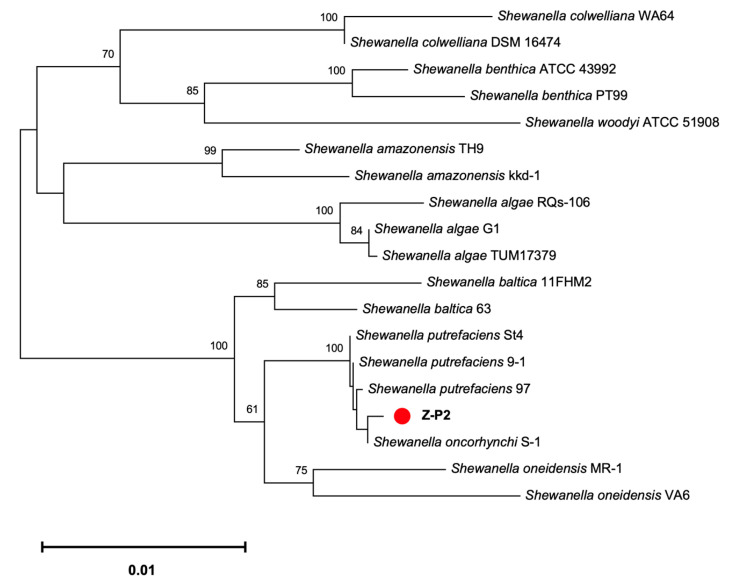
Neighbor-joining tree with a bootstrap test (1000 replicates) based on the 16S rDNA gene sequences of *S. oncorhynchi* Z-P2. The scale bar indicates the evolutionary distance in nucleotide substitutions per site.

The raw sequencing data from the Nanopore PromethION platform included 268,526 reads for 1,365,575,589 bases, and the raw sequencing depth was 277.32×. The complete genome of *S. oncorhynchi* Z-P2 is composed of a single circular chromosome of 5,034,612 bp with a G + C content of 45.4% (Figure 1). In addition, 4544 coding DNA sequences (CDSs), 109 tRNAs, 31 rRNAs (11 5s rRNAs, 10 16s rRNAs and 10 23s rRNAs) and 0 sRNA were predicted. The annotations by RAST reported a total of 1803 functional genes of subsystems in Figure 2. The main subsystems were related to amino acids and derivatives (312 genes), followed by protein metabolism (244 genes), carbohydrates (191 genes), cofactors, vitamins, prosthetic groups, pigments (151 genes), membrane transport (134 genes) and respiration (115 genes). For KEGG pathway annotation, all 3835 genes were classified in 220 KEGG pathways and the level 1 and level 2 pathways were shown in Figure 3. The analysis revealed that amino acid metabolism (193 genes), carbohydrate metabolism (186 genes), metabolism of cofactors and vitamins (173 genes), signal transduction (161 genes), energy metabolism (137 genes) and membrane transport (107 genes) were obviously enriched. Among these pathways, the two-component system (map02020, 143 genes), purine metabolism (map00230, 75 genes), ABC transporters (map02010, 69 genes), pyrimidine metabolism (map00230, 54 genes), ribosome (map03010, 53 genes), bacterial chemotaxis (map02030, 52 genes) and so on were mapped on level 3 pathways. Moreover, the phylogenetic tree based on the 16S rDNA gene sequences showed that Z-P2 was assigned to the *S. oncorhynchi* (Figure 4).

The whole genome of *S. oncorhynchi* Z-P2 consists of eight genomic islands (Table S2) and no virulence factors and antibiotic resistance genes were found in the islands. The distribution of the genes in genomic islands were transporters, recombinase and phage-derived protein. A total of 30 putative virulence factor genes passing an identify threshold of 70% were predicted in the genome of Z-P2 (Table S3). These virulence factors were involved with the inflagellar-related protein, chemotaxis protein, quorum sensing signal and ferric uptake regulator. In addition, Z-P2 possessed five CRISPR arrays and three different sets of *cas* genes (Table 1). One annotated CRISPR-CAS system existent in Z-P2 belongs to the subtype I-Fv, encoded by *cas7f* and *cas5f* genes [35]. The CRISPR-CAS system is an adaptive immune mechanism against bacteriophages and invasive nucleic acids in the bacterial genome [36].

**Figure 5:**
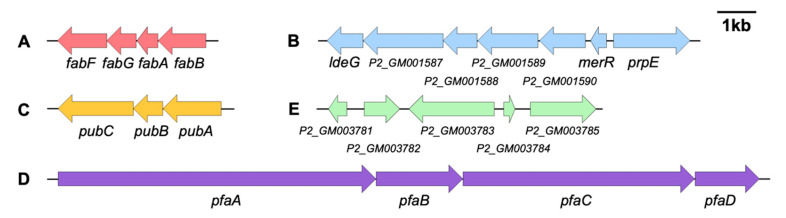
The secondary metabolite biosynthetic gene clusters identified in *S. oncorhynchi* Z-P2. (**A**): APE biosynthetic gene cluster; (**B**): beta-lactone biosynthetic gene cluster; (**C**): putrebactin biosynthetic gene cluster; (**D**): eicosapentaenoic acid biosynthetic gene cluster; (**E**): RiPP biosynthetic gene cluster.

Five NRPS and polyketide synthetase (PKS) gene clusters were identified in the genome of *S. oncorhynchi* Z-P2, including an aryl polyene (APE) biosynthetic gene cluster (location: 380,670 to 384,522) [37], a beta-lactone biosynthetic gene cluster (location: 1,709,748 to 1,719,319) [38], a siderophore type gene cluster (location: 1,858,771 to 1,862,960) synthesizing putrebactin [15], a ghlE-KS type gene cluster (location: 3,385,779 to 3,403,945) synthesizing eicosapentaenoic acid (EPA) [39] and a ribosomally synthesized and post-translationally modified peptide (RiPP) biosynthetic gene cluster (location: 4,156,609 to 4,163,174) (Figure 5).

### 3.2. Genome Synteny and Pan-Genome Analysis

**Figure 6:**
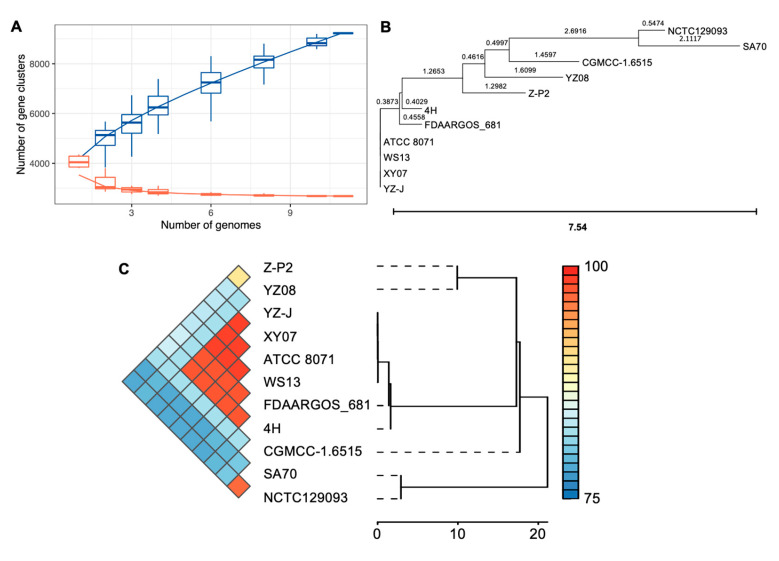
Pan-genome analysis of *S. oncorhynchi* Z-P2 and 10 *S. putrefaciens* strains. (**A**) The number of pan-gene clusters (blue) and core gene clusters (orange) among Z-P2 and different *S. putrefaciens* strains. (**B**) Phylogenetic tree of Z-P2 and 10 *S. putrefaciens* strains based on whole genomes. (**C**) Heatmap and hierarchical clustering based on pairwise average nucleotide identity (ANI) values.

**Figure 7:**
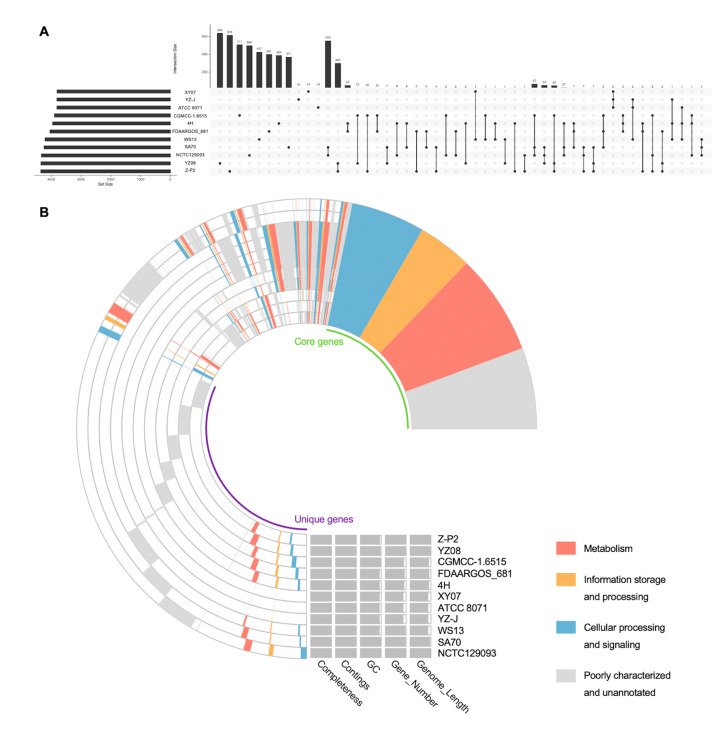
Distribution of genes among *S. oncorhynchi* Z-P2 and 10 *S. putrefaciens* strains. (**A**) Upset plot of comparisons among unique genes of Z-P2 and *S. putrefaciens* strains. (**B**) Pan-genome profile of Z-P2 and *S. putrefaciens* strains. COG annotation showing the core genes and unique genes of Z-P2 and *S. putrefaciens* strains.

*S. oncorhynchi* is a novel species of the genus *Shewanella* and its complete genome sequence has never been reported [14]. Therefore, ten genomes of known *S. putrefaciens* (closely related species of *S. oncorhynchi*) strains and Z-P2 were investigated for a genome synteny and pan-genome analysis. As shown in Figure 6A, a total of 9228 pan-gene clusters were identified, and core gene clusters decreased to 2681 as the number of genomes included increased. The genomic phylogenetic tree analysis showed that Z-P2 was most closely related to the strain YZ08 (Figure 6B). The relationships within the species were further refined by the heatmap and hierarchical clustering tree of ANI values (Figure 6C). The ANI value between Z-P2 and YZ08 was the highest (90.09%) of the strains. In addition, the genome synteny analysis revealed that Z-P2 and YZ08 shared many homologous regions, while a lot of genomic rearrangements were found (Figure S1). The upset plot showed that the unique gene clusters in each genome were in the range of 24–640 (Figure 7A). Compared with the other 10 strains, 618 unique gene clusters were identified in Z-P2 and 554 gene clusters were shared with the most closely related strain, YZ08. The pan-genome profile based on COG annotation revealed that the core gene clusters included 921 for metabolism, 503 for information storage and processing and 682 for cellular processes and signaling (Figure 7B). Additionally, the unique gene clusters of Z-P2 included 73 for metabolism, 25 for information storage and processing and 35 for cellular processes and signaling. In COG distribution, an iron uptake system gene cluster was identified in the unique gene clusters of Z-P2, annotated as COG0735 (Fe^2+^ or Zn^2+^ uptake regulation protein Fur/Zur), which may play an important role in iron uptake and the regulation of siderophore biosynthesis [4,40].

### 3.3. Analysis of the Putrebactin Biosynthetic Gene Cluster and Exporters Genes

**Figure 8:**
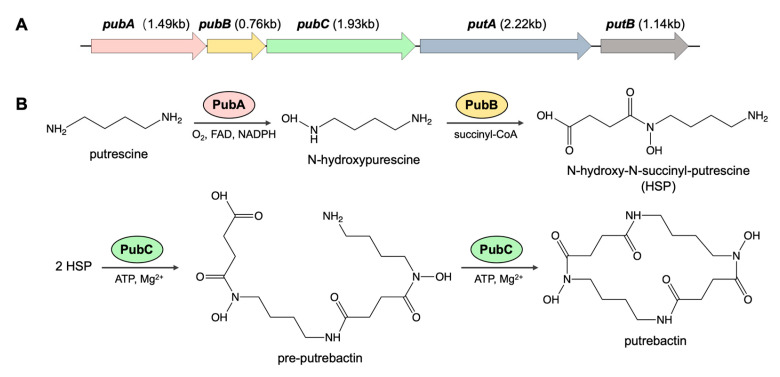
Putrebactin biosynthesis gene cluster (**A**) and proposed biosynthetic pathway (**B**) of *S. oncorhynchi* Z-P2.

The complete putrebactin biosynthetic gene cluster was identified in the genome of *S. oncorhynchi* Z-P2 and the biosynthetic mechanism of putrebactin was analyzed. This gene cluster contains three core biosynthesis genes *pubA*–*C* followed by two trailing genes *putAB* with the function of siderophore-mediated iron uptake (Figure 8A) [4,15]. The first gene of the putrebactin operon is *pubA*, which encodes an amine monooxygenase of 56.34 kDa. The second gene is *pubB*, which encodes a N-hydroxydiamine-succinyl CoA transferase of 27.36 kDa [41]. The *pubC* encodes a IucC-like NIS synthetase of 73.29 kDa [3,15]. Finally, operon *putAB* encodes a TonB-dependent siderophore receptor (TBSR) and a ferrisiderophore reductase (FSR), respectively [4]. In addition, the comparative analysis of putrebactin synthetase gene clusters from different strains in *Shewanella* illustrated high identity (Figure S2).

The biosynthesis pathways of putrebactin are catalyzed by the PubABC multienzyme system, which assembles putrebactin from putrescine (Figure 8B). Putrescine is derived from the reactions catalyzed by ornithine decarboxylase (ODC) SpeF using L-ornithine as the specific substrate in *Shewanella* [42,43]. For the PubABC system, PubA could catalyze the O_2_- and FAD-dependent hydroxylation of putrescine to form N-hydroxypurescine. And PubB could catalyze succinyl-CoA-dependent succinylation of N-hydroxypurescine to form N-hydroxy-N-succinyl-putrescine (HSP). Finally, in a two-step catalytic mechanism, PubC could catalyze ATP-dependent head-to-tail dimerization of HSP to form pre-putrebactin, and subsequent macrocyclization could form putrebactin, which is a macrocyclic dimer of HSP [15].

Additionally, the genes of exporters for the release of siderophores were predicted in the genome of Z-P2 (Table 2). These exporters involved the ABC transporter superfamily, the major facilitator superfamily (MFS) and the resistance-nodulation-division (RND) superfamily [44].

### 3.4. Identification of Putrebactin

**Figure 9:**
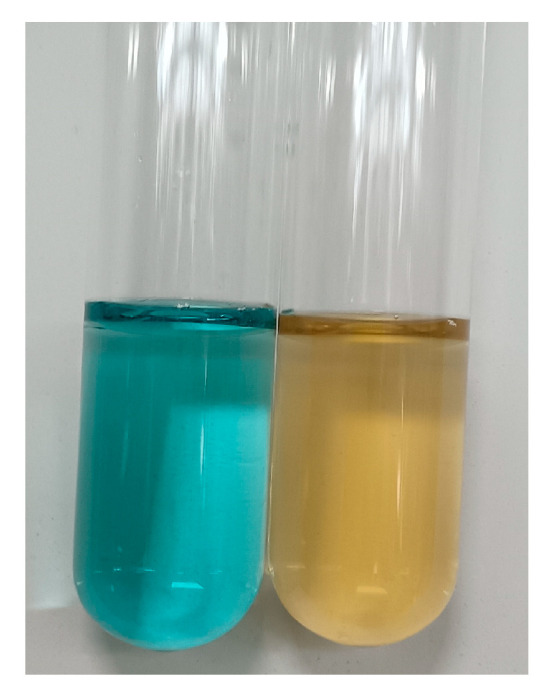
Siderophore production of *S. oncorhynchi* Z-P2. **Left**: CAS solution and uninoculated broth. **Right**: CAS solution and cell-free supernatant of Z-P2.

**Figure 10:**
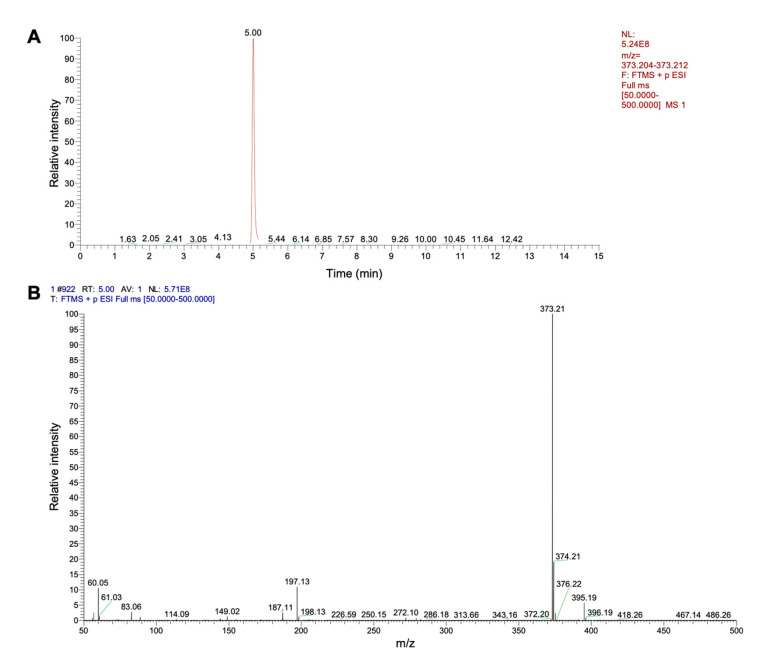
Putrebactin production of *S. oncorhynchi* Z-P2. (**A**) Base peak chromatogram (BPC). (**B**) Mass spectrum.

To confirm the production of putrebactin by *S. oncorhynchi* Z-P2, the culture of this strain was detected by CAS assay and mass spectrometry. As shown in Figure 9, the presence of orange CAS solution with cell-free supernatant indicated siderophore production by Z-P2. Siderophores perform competitive ferric iron chelation from CAS–iron complex and then CAS dye becomes free in the reaction solution with the color change from blue to orange [34]. The siderophore production units of Z-P2 were measured to be 42.08 ± 0.66%. From crude extract of Z-P2 culture, one type of siderophore, putrebactin (C_16_H_28_O_6_N_4_), was detected by UPLC-MS (retention time = 5.00 min) (Figure 10). Putrebactin was found to have an [M + H]^+^ molecular ion at *m*/*z* 373.21, consistent with previous reports [15,43]. This result was consistent with the putrebactin biosynthetic gene cluster analysis. Putrebactin was the first siderophore to be identified in *Shewanella* species [45], then later it was discovered that bisucaberin and avaroferrin were produced by *Shewanella algae* B516 [46]. Like putrebactin, bisucaberin and avaroferrin biosynthesized by genes *pubA*–*C* also belong to the dimeric macrocyclic class of siderophore [43]. However, bisucaberin and avaroferrin were not detected in the Z-P2 culture. Due to the low secretion level, bisucaberin and avaroferrin with precursor (cadaverine) supplementation can be detected in *Shewanella* culture [43,47].

## 4. Conclusions

In this study, the whole genome of the siderophore putrebactin-producing *S. oncorhynchi* Z-P2 was sequenced and analyzed. The whole genome consists of a circular chromosome of 5,034,612 bp with a G + C content of 45.4%, which is the first report of whole genome information of *S. oncorhynchi*. The core and strain-unique gene clusters of Z-P2 and *S. putrefaciens* genomes were investigated, which showed obvious differences in their genomic signatures. Compared with the other *S. putrefaciens* strains, 618 unique gene clusters, including 73 for metabolism, 25 for information storage and processing and 35 for cellular processes and signaling, were identified in Z-P2. Especially, an Fe^2+^ uptake regulation protein Fur, which may play an important role in iron uptake and regulation of siderophore biosynthesis, was identified. Moreover, the gene cluster involved in putrebactin biosynthesis in Z-P2 was annotated, and the mechanism of putrebactin biosynthesis was analyzed. Analysis of the genetic and functional characteristics of Z-P2 demonstrated its capacity to produce putrebactin. These findings have important implications for the understanding of the bacterial siderophore biosynthetic mechanism, particularly putrebactin produced by *Shewanella*. It also offers support for potential applications in drug production and further molecular biology research on related genes.

**Genome Sequence Accession Number:** The complete genome sequence *Shewanella oncorhynchi* Z-P2 has been deposited at GenBank under the accession number CP132914.

## Figures and Tables

**Table 1:** CRISPR arrays and *cas* genes of the *S. oncorhynchi* Z-P2 genome.

CRISPR Array Location	Length (bp)	Spacer Length	Spacer Numbers	DR ^1^ Length	*cas* Genes
1,457,061–457,246	186	54	2	26	-
911,996–912,258	263	33	3	40	-
1,238,331–1,244,119	5789	31	96	28	*cas1f* (1,231,745–1,232,722) *cas3* (1,232,735–1,235,671) *cas7fv* (1,235,675–1,236,622) *cas5fv* (1,236,631–1,237,641) *cas6f* (1,237,650–1,238,201)
2,074,007–2,075,908	1902	32	27	36	-
2,087,585–2,089,626	2042	32	29	36	*cas2* (2,076,073–2,076,216) *cas6* (2,077,318–2,078,265) csx3 (2,078,361–2,078,699) RA178_09445 (2,081,075–2,081,731) RA178_09450 (2,081,728–2,083,311) RA178_09455 (2,083,308–2,084,741) RA178_09465 (2,085,256–2,087,445)

^1^ DR: direct repeat sequences.

**Table 2:** Putative siderophore exporter genes of the *S. oncorhynchi* Z-P2 genome.

Type	Gene Location	Length (bp)	Gene ID	Product
ABC ^1^	844,226–846,259	2034	RA178_03930	MacB family efflux pump subunit
MFS ^2^	1,302,942–1,304,105	1164	RA178_05870	multidrug effflux MFS transporter
	2,333,160–2,334,383	1224	RA178_10585	multidrug effflux MFS transporter
	2,484,985–2,486,217	1233	RA178_11215	multidrug efflux MFS transporter EmrD
	4,783,149–4,784,357	1209	RA178_21055	multidrug effflux MFS transporter
RND ^3^	675,824–678,895	3072	RA178_03090	efflux RND transporter permease subunit
	696,040–699,126	3087	RA178_03150	efflux RND transporter permease subunit
	842,991–844,223	1233	RA178_03925	efflux RND transporter permease subunit
	1,075,848–1,077,110	1263	RA178_04820	HlyD family efflux transporter periplasmic adaptor subunit
	1,291,279–1,294,437	3159	RA178_05825	multidrug efflux RND transporter permease subunit
	1,304,185–1,307,325	3141	RA178_05875	efflux RND transporter permease subunit
	1,559,138–1,562,218	3081	RA178_07075	efflux RND transporter permease subunit
	1,782,382–1,785,630	3249	RA178_08120	efflux RND transporter permease subunit
	2,047,189–2,050,284	3096	RA178_09240	efflux RND transporter permease subunit
	2,146,014–2,149,133	3120	RA178_09810	multidrug efflux RND transporter permease subunit
	2,169,901–2,172,948	3048	RA178_09915	efflux RND transporter permease subunit
	2,172,945–2,176,196	3252	RA178_09920	efflux RND transporter permease subunit
	3,275,726–3,276,736	1011	RA178_14795	HlyD family efflux transporter periplasmic adaptor subunit
	4,005,831–4,009,022	3192	RA178_17790	efflux RND transporter permease subunit
	4,364,416–4,367,640	3225	RA178_19380	efflux RND transporter permease subunit
	4,828,489–4,831,632	3144	RA178_21255	efflux RND transporter permease subunit
	4,905,088–4,908,222	3135	RA178_21580	efflux RND transporter permease subunit
	4,929,711–4,932,878	3168	RA178_21675	efflux RND transporter permease subunit
	4,944,067–4,947,120	3054	RA178_21735	CusA/CzcA family heavy metal efflux RND

^1^ ABC: ABC transporter superfamily. ^2^ MFS: major facilitator superfamily. ^3^ RND: resistance-nodulation-division superfamily.

## Data Availability

Data are contained within the article or Supplementary Materials.

## Supplementary Materials

The following supporting information can be downloaded at: https://www.mdpi.com/article/10.3390/microorganisms11122961/s1, Figure S1: Genome synteny analysis of *Shewanella oncorhynchi* Z-P2 and *S. putrefaciens* strains; Figure S2: Comparative analysis with putrebactin biosynthetic gene clusters of *Shewanella* strains; Table S1: List of *S. putrefaciens* genomes analyzed; Table S2: Details of the genomic islands of the *S. oncorhynchi* Z-P2 genome; Table S3: The virulence factor genes of the *S. oncorhynchi* Z-P2 genome.
