# Supplementary material for: Complete Genome Sequence and Pan-Genome Analysis of Shewanella oncorhynchi Z-P2, a Siderophore Putrebactin-Producing Bacterium
Source: Microorganisms. 2023 Dec 11;11(12):2961. doi: 10.3390/microorganisms11122961 (PMC10745600; doi:10.3390/microorganisms11122961)
Supplement: Supplementary file 1 [file microorganisms-11-02961-s001.zip › microorganisms-2684436-supplementary.pdf]

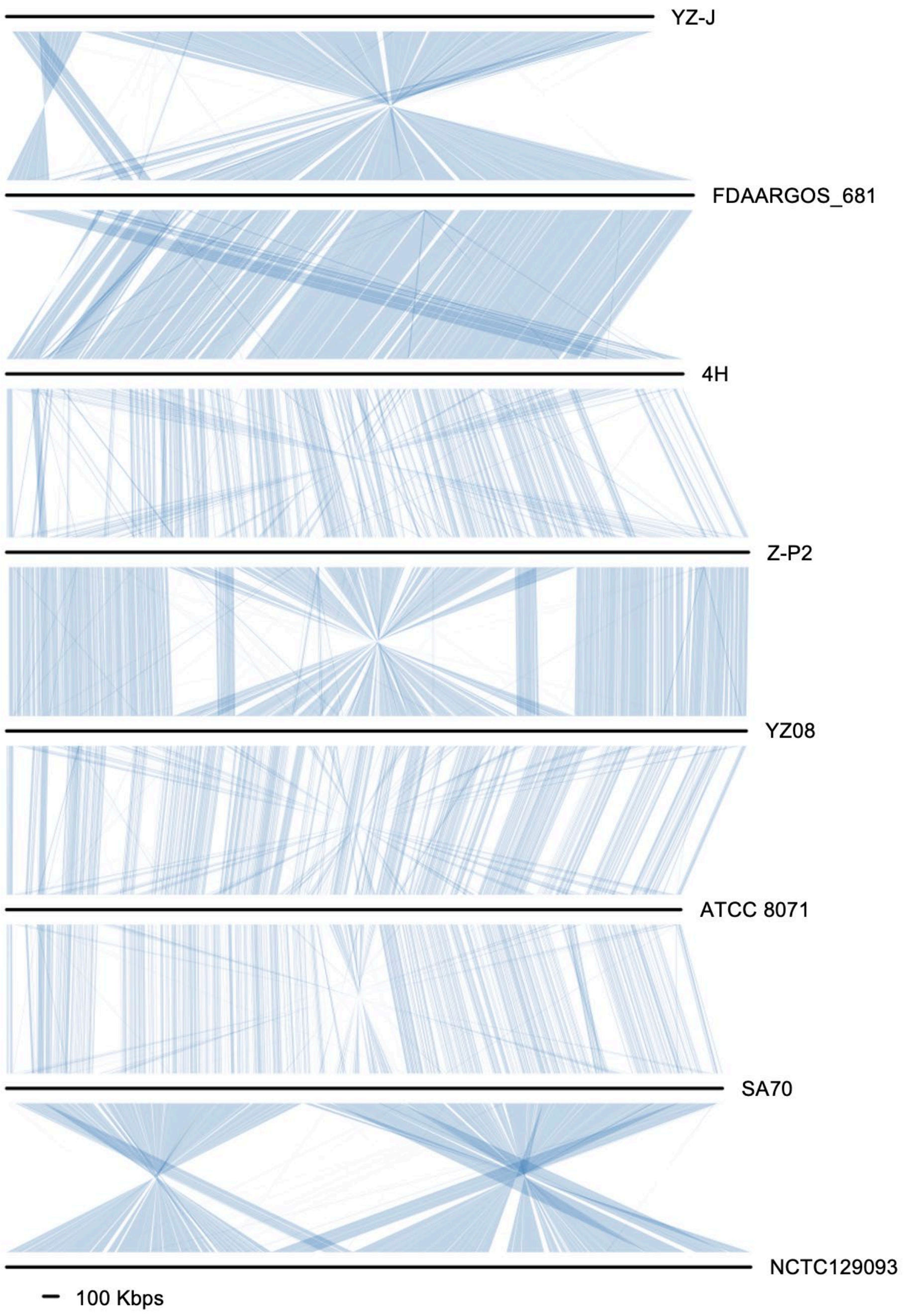

Figure S1. Genome synteny analysis of *Shewanella oncorhynchi* Z-P2 and *S. putrefaciens* strains. Collinear blocks between the two strains are linked by lines.

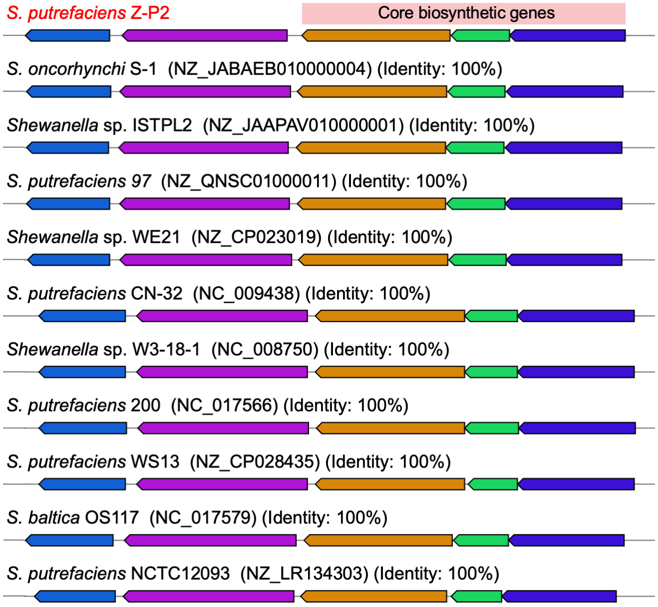

Figure S2. Comparative analysis with putrebactin biosynthetic gene clusters of *Shewanella* strains.

Table S1. List of *S. putrefaciens* genomes analyzed.

No.	Strain	NCBI accession	Genome size (Mb)	GC (%)
1	4H	GCA_025402875.1	4.6	44.5
2	ATCC 8071	GCA_016406325.1	4.4	44
3	NCTC129093	GCA_900636665.1	5	47.5
4	YZ08	GCA_019599085.1	5.1	46
5	SA70	GCA_002157365.2	5.3	47.5
6	FDAARGOS_681	GCA_009730575.1	4.7	44
7	CGMCC-1.6515	GCA_016406305.1	4.6	47
8	XY07	GCA_017068195.1	4.4	44
9	YZ-J	GCA_019599125.1	4.4	44
10	WS13	GCA_003044255.1	4.4	44

Table S2. Details of the genomic islands of the *S. oncorhynchi* Z-P2 genome.

Island location	Length (bp)	GC%	Gene numbers	Gene ID	Product
806,970-815,269	8,300	43.84	7	RA178_03755, RA178_03760, RA178_03765, RA178_03770, RA178_03775, RA178_03780, RA178_03785	site-specific integrase
1,370,953-1,416,822	45,870	38.54	49	RA178_06125, RA178_06130, RA178_06135, RA178_06140, RA178_06145, RA178_06150, RA178_06155, RA178_06160, RA178_06165, RA178_06170, RA178_06175, RA178_06180, RA178_06185, RA178_06190, RA178_06195, RA178_06200, RA178_06205, RA178_06210, RA178_06215, RA178_06220, RA178_06225, RA178_06230, RA178_06235, RA178_06240, RA178_06245, RA178_06250, RA178_06255, RA178_06260, RA178_06265, RA178_06270, RA178_06275, RA178_06280, RA178_06285, RA178_06290, RA178_06295, RA178_06300, RA178_06305, RA178_06310, RA178_06315, RA178_06320, RA178_06325, RA178_06330, RA178_06335, RA178_06340, RA178_06345, RA178_06350, RA178_06355, RA178_06360, RA178_06365, RA178_06370, RA178_06375, RA178_06380	carbon storage regulator CsrA, tRNA-Ser, tRNA-Arg, KilA-N domain-containing protein, helix-turn-helix domain-containing protein, DNA-directed RNA polymerase, P27 family phage terminase small subunit, terminase large subunit, phage portal protein, phage major capsid protein, head-tail connector protein, tail fiber domain-containing protein, valine--tRNA ligase
2,068,129-2,094,117	25,989	47.2	24	RA178_09370, RA178_09375, RA178_09380, RA178_09385, RA178_09390, RA178_09395, RA178_09400, RA178_09405, RA178_09410, RA178_09415, RA178_09420, RA178_09425, RA178_09430, RA178_09435, RA178_09440, RA178_09445, RA178_09450, RA178_09455, RA178_09460, RA178_09465,	helix-turn-helix domain-containing protein, replication protein P, tyrosine-type recombinase/integrase, helix-turn-helix transcriptional regulator, CRISPR-associated endonuclease Cas2, CRISPR system precrRNA processing endoribonuclease RAMP protein Cas6, CRISPR-associated protein Csx3, RAMP superfamily CRISPR-associated protein, SOS

				RA178_09470, RA178_09475, RA178_09480, RA178_09485	response-associated peptidase family protein
2,103,837-2,117,630	13,794	46.42	22	RA178_09535, RA178_09540, RA178_09545, RA178_09550, RA178_09555, RA178_09560, RA178_09565, RA178_09570, RA178_09575, RA178_09580, RA178_09585, RA178_09590, RA178_09595, RA178_09600, RA178_09605, RA178_09610, RA178_09615, RA178_09620, RA178_09625, RA178_09630, RA178_09635, RA178_09640	DGQHR domain-containing protein, helix-turn-helix transcriptional regulator, Cro/CI family transcriptional regulator, helix-turn-helix domain-containing protein, replication protein P, tyrosine-type recombinase/integrase
2,709,139-2,742,614	33,476	39.62	27	RA178_12230, RA178_12235, RA178_12240, RA178_12245, RA178_12250, RA178_12255, RA178_12260, RA178_12265, RA178_12270, RA178_12275, RA178_12280, RA178_12285, RA178_12290, RA178_12295, RA178_12300, RA178_12305, RA178_12310, RA178_12315, RA178_12320, RA178_12325, RA178_12330, RA178_12335, RA178_12340, RA178_12345, RA178_12350, RA178_12355, RA178_12360	site-specific integrase, HDOD domain-containing protein, tyrosine-type recombinase/integrase, helix-turn-helix transcriptional regulator, IS3 family transposase, Na^+/H^+ antiporter, transposase, amidohydrolase family protein, FimD/PapC C-terminal domain-containing protein, fimbria/pilus outer membrane usher protein, molecular chaperone, transporter substrate-binding domain-containing protein, response regulator transcription factor, EAL domain-containing response regulator
2,744,216-2,756,057	11,842	42.93	10	RA178_12375, RA178_12380, RA178_12385, RA178_12390, RA178_12395, RA178_12400, RA178_12405, RA178_12410, RA178_12415, RA178_12420	thioredoxin family protein, thioredoxin family protein, metalloregulator ArsR/SmtB family transcription factor, transposase, linear amide C-N hydrolase, aspartate-alanine antiporter, glutamate decarboxylase, IS3 family transposase, TolC family protein, FUSC family protein, multidrug transporter subunit MdtN
3,382,746-3,403,945	21,200	49.82	7	RA178_15260, RA178_15265, RA178_15270, RA178_15275, RA178_15280, RA178_15285, RA178_15290	4'-phosphopantetheinyl transferase superfamily protein, transcriptional regulator, beta-ketoacyl synthase N-terminal-like, PfaB family protein, eicosapentaenoate synthase subunit PfaD

4,592,104-4,603,039	10,936	38.26	7	RA178_20255, RA178_20260, RA178_20265, RA178_20270, RA178_20275, RA178_20280, RA178_20285	DDE-type integrase/transposase/recombinase, TniB family NTP-binding protein, TniQ family protein, UvrD-helicase domain-containing protein, WYL domain-containing protein, DUF2220 family protein

Table S3. The virulence factors genes of the *S. oncorhynchi* Z-P2 genome.

Gene location	Length (bp)	Identity	VFDB ID	VF ID	VF name	Related gene
5,645-7,462	1,818	98.5	VFG041976	SS008	T3SS	RSp1236 hypothetical protein
175,466-177,031	1,566	70.1	VFG038278	CVF780	T2SS	*exeE* general secretion pathway protein E
178,321-178,755	435	78.3	VFG038284	SS209	exe	*exeG* general secretion pathway protein G
230,308-231,492	1,185	81.9	VFG046474	CVF827	EF-Tu	*OOM_0626* elongation factor
247,014-248,252	1,239	81.7	VFG046474	CVF827	EF-Tu	*OOM_0626* elongation factor
480,125-481,099	975	75.3	VFG013197	CVF506	Heme biosynthesis	*hemB* Porphobilinogen synthase
703,272-704,909	1,638	73.3	VFG001855	VF0159	Hsp60	*htpB* Hsp60, 60K heat shock protein HtpB
1,360,983-1,361,963	981	81.3	VFG000477	VF0112	RpoS	*rpoS* sigma S (sigma 38) factor of RNA polymerase, major sigmafactor during stationary phase
1,370,953-1,371,150	198	77	VFG010906	CVF362	Carbon storage regulator A	*csrA* global regulator (carbon storage regulator)
1,503,467-1,504,504	1,038	80.8	VFG038466	CVF783	Tap type IV pili	*tapT* Pilus retraction ATPase PilT
1,588,990-1,589,910	921	70.7	VFG038817	CVF786	Polar flagella	*cheV* two-component system chemotaxis response regulator CheV
1,591,398-1,591,814	417	74.6	VFG038803	AI144	ND	*flgC* flagellar basal body rod protein FlgC
1,613,132-1,614,184	1,053	77.1	VFG038633	CVF786	Polar flagella	*fliG* flagellar motor switch protein FliG
1,615,135-1,616,472	1,338	71.7	VFG038641	CVF786	Polar flagella	*fliI* flagellar protein export ATPase FliI
1,619,725-1,620,753	1,029	78.2	VFG038656	CVF786	Polar flagella	*fliM* flagellar motor switch protein FliM
1,620,765-1,621,145	381	81.7	VFG038660	CVF786	Polar flagella	*fliN* polar flagellar switch protein FliN
1,624,769-1,626,868	2,100	70.8	VFG038685	CVF786	Polar flagella	*flhA* flagellar biosynthesis protein FlhA
1,628,267-1,629,148	882	74.4	VFG038691	VF0473	Polar flagella	*flhG* flagellar-related protein
1,629,914-1,630,285	371	92.6	VFG007568	VF0519	Flagella	*cheY* chemotaxis protein CheY
1,636,833-1,637,327	495	80.1	VFG043204	AI144	ND	*cheW-2* chemotaxis protein CheW

1,650,810-1,651,889	1,080	72.9	VFG037923	CVF775	Capsule	*M3Q_285* nucleoside-diphosphate sugar epimerase
1,651,966-1,652,841	876	76.2	VFG037938	CVF775	Capsule	*M3Q_286* bifunctional UDP-N-acetylglucosamine pyrophosphorylase
1,662,978-1,664,144	1,167	77.3	VFG048789	CVF854	Capsule	*A225_3877* UDP-glucose dehydrogenase
2,190,392-2,190,823	432	73	VFG000478	VF0113	Fur	*fur* transcriptional repressor of iron-responsive genes (Fur family) (ferric uptake regulator)
3,067,432-3,068,178	747	76.2	VFG038840	VF0473	Polar flagella	*flmH* flagellar-related 3-oxoacyl-ACP reductase
3,487,184-3,487,951	768	71.4	VFG007601	CVF281	Flagella	*motA* flagellar motor protein
3,788,422-3,790,995	2,574	77.1	VFG049190	CVF861	T6SS-II	*KOX_00005* protein disaggregation chaperone
3,890,242-3,890,712	471	78.2	VFG018243	CVF628	AI-2	*luxS* S-ribosylhomocysteinase
4,099,459-4,100,307	849	70.9	VFG013465	CVF494	LOS	*kdsA* 2-dehydro-3-deoxyphosphooctonate aldolase
4,448,306-4,449,370	1,065	74.6	VFG013200	CVF506	Heme biosynthesis	*hemE* uroporphyrinogen decarboxylase
